# Ductal closure using the Amplatzer duct occluder type two: experience in Port Elizabeth hospital complex, South Africa

**DOI:** 10.5830/CVJA-2013-033

**Published:** 2013-07

**Authors:** Lungile Pepeta, Adele Dippenaar

**Affiliations:** Division of Paediatric Cardiology, Paediatrics and Child Health, Dora Nginza Hospital, Port Elizabeth, Eastern Cape South Africa; Division of Paediatric Cardiology, Paediatrics and Child Health, Dora Nginza Hospital, Port Elizabeth, Eastern Cape South Africa

**Keywords:** patent ductus arteriosus, Amplatzer duct occluder II, percutaneous ductal closure

## Abstract

**Objective:**

To report outcomes in percutaneous ductal closure using the Amplatzer duct occluder type two (ADO II).

**Methods:**

Records of patients admitted for percutaneous closure of patent ductus arteriosus (PDA) were reviewed.

**Results:**

From May 2009 to July 2012, 36 patients were assigned to closure using the ADO II. There were 21 females and 15 males. The median age was 16.5 (2–233) months; median weight, 8 (3.94–39.2) kg; and median height, 75 (55–166) cm. The mean pulmonary artery pressure was 24.4 (± 10.4) mmHg, the pulmonary blood flow:systemic blood flow (Qp:Qs) ratio was 2.25 (± 1.97), and mean pulmonary resistance (Rp) was 1.87 (± 1.28) Wood units. The mean ductal size was 2.74 (± 1.3) mm. In 30 patients the device was delivered through the pulmonary artery. Thirty-three patients achieved complete closure by discharge (day one).

**Conclusion:**

The ADO II is capable of closing a wide range of ducts in carefully selected patients. Our findings are comparable with other studies regarding ductal closure rates.

## Abstract

The incidence of patent ductus arteriosus (PDA) accounts for 11.9 to 15.6% of all congenital heart diseases.^1^,^2^ This figure rises to about 31% in premature infants.^3^ Surgical closure of the PDA was first reported by Gross, *et al.* in 1938.^4^ However, it was not until 1967 when Porstmann, *et al.* reported the first percutaneous closure of the PDA in the cardiac catheterisation laboratory.^5^ Several devices have been introduced for transcatheter closure of the PDA over the years.^6^-^16^ In 2008, the Amplatzer duct occluder type two (ADO II) (St Jude Medical, Cardiovascular Division, St Paul, MN) was introduced.^17^ We report on our experience from a single centre.

## Methods

Following ethics clearance, a review of records of patients who underwent percutaneous closure of the PDA in the Port Elizabeth Provincial Hospital, Port Elizabeth, South Africa was performed. Patients’ age, gender, weight, pulmonary blood flow:systemic blood flow (Qp:Qs) ratios, and pulmonary resistance (Rp) were documented. Angiographic anatomy, including narrowest diameter (ductal size), ductal length and ductal ampulla; selection of ductal closure device; ductal closure approach; radiological screening time; complications and outcomes were also noted.

**Fig. 1.**
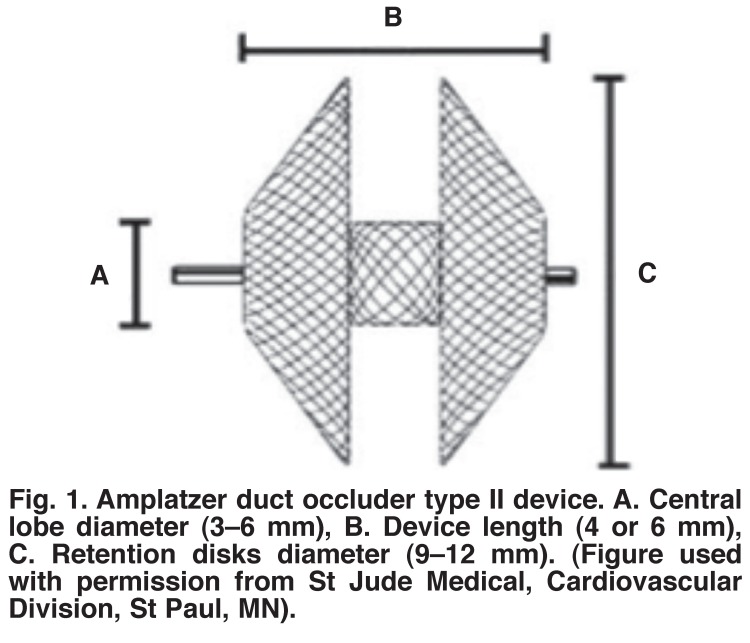
Amplatzer duct occluder type II device. A. Central lobe diameter (3–6 mm), B. Device length (4 or 6 mm), C. Retention disks diameter (9–12 mm). (Figure used with permission from St Jude Medical, Cardiovascular Division, St Paul, MN).

The Amplatzer duct occluder type two device is made of a meshwork of self-expandable nitinol wire. It consists of a central ‘lobe’, which measures 3–6 mm in diameter, and two retention disks on either side of the lobe (Fig. 1). The disks are 6 mm larger than the central lobe and range from 9–12 mm in diameter. The devices are designed in such a way that the central lobe is the one that is placed in the duct itself, with a retention disk on either side of the PDA.

**Fig. 2.**
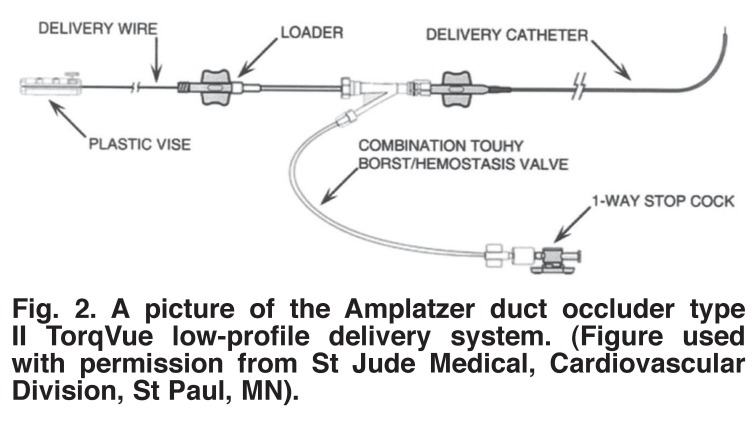
A picture of the Amplatzer duct occluder type II TorqVue low-profile delivery system. (Figure used with permission from St Jude Medical, Cardiovascular Division, St Paul, MN).

The device is delivered using a TorqVue low-profile (LP) delivery system (Fig. 2). The reason these newer devices can be delivered using a low-profile delivery system is that they lack the polyester material that is present in the Amplatzer duct occluder type one (ADO I) (St Jude Medical, Cardiovascular Division, St Paul, MN). The delivery system has delivery sheaths of 4- and 5-F in size, with a length of either 60 or 80 cm; delivery wire with a screw mechanism to attach the device; device loader; Y-connector and a plastic vise.

Informed consent is obtained before attempting percutaneous ductal closure. Under conscious sedation, the patient is scrubbed and draped to ensure a sterile environment. Femoral arterial and venous access is achieved using standard vascular-access short sheaths. About 50 IU/kg of heparin are then given through the arterial sheath. Descending aortography in the straight lateral view is performed.

The size and shape (type) of the PDA using the Krichenko classification are determined.^18^ Standard left and right cardiac catheterisation procedures are then performed. Calculations to ascertain the extent of left-to-right (or right-to-left) shunting, pulmonary vascular and systemic vascular resistances are done.

Following angiography and haemodynamic data, the decision whether or not to close the PDA is then made. If the PDA is amenable to percutaneous closure based on the size and length of the duct, an appropriate device is selected using the manufacturer’s device selection table (St Jude Medical, Cardiovascular Division, St Paul, MN) as a guide (Table 1). The delivery system is then flushed using heparinised saline.

**Table 1:** Manufacturer’s Guidelines Regarding ADO II Device Size Choice In Relation To The PDA Size And Length

	*Ductal length*			
*Ductal size*	*< 5 mm*	*5.1–8 mm*	*8.1–10 mm*	*10.1–11 mm*
< 2.5 mm	3 × 4	3 × 6	4 × 6	5 × 6
2.5–3.5 mm	4 × 4	4 × 6	5 × 6	6 × 6
3.6–4.5 mm	5 × 4	5 × 6	5 × 6	6 × 6
4.6–5.5 mm	6 × 4	6 × 6	6 × 6	6 × 6

A 0.035-inch guide wire is passed across the PDA using an end-hole catheter, either in an anterograde fashion through the pulmonary side or in a retrograde manner via the aortic route. The ADO II delivery sheath is passed across the PDA over the guide wire. Blood is allowed to flow from the back of the sheath to purge all air from the system. The delivery wire is passed through the loader. The device is attached to the delivery wire using a screw mechanism.

Under water, the device is retrieved into the loader so that its distal radiopague end is at the tip of the loader. The loader is firmly introduced into the delivery sheath. Under fluoroscopy, the device is advanced into the sheath using the delivery wire until it reaches the tip of the delivery sheath. At this stage the whole assembly is repositioned until the operator is satisfied, to deploy the distal disk.

Once the distal disk is well positioned and conforms to the vessel wall, the middle lobe is deployed in the duct with the proximal disk deployed on the other end of the PDA. Angiography may be performed at any stage of device deployment using the Y-connector and an angiographic catheter to check for device positioning in the duct, pulmonary artery or aorta. The device is released or retrieved as the operator deems fit.

The patient receives an intravenous antibiotic and may receive prophylaxis for infective endocarditis for six months. The patient is followed up at one day, one month, three months, six months, one year and two years following transcatheter closure of the PDA, using this device, to look for complications that may arise from the catheterisation procedure or the device itself. After two years’ follow up, patients are discharged.

Complications relating to closure of PDA in our patients, including aortic and (left) pulmonary obstruction, and device embolisation are documented. Short-term outcomes are also reported.

## Statistical analysis

Values were reported as mean ± standard deviation (SD) and median (range). Statistical significance was not required, as data comparison was not done.

## Results

Between May 2009 and July 2012, 36 patients were selected for percutaneous closure of the PDA using the Amplatzer duct occluder II. Their median age was 16.5 months (range: 2–233), with a median weight of 8 kg (range: 3.9–39.2), and a median height of 75 cm (range: 55–166). There were 21 females and 15 males. Patients’ basic characteristics and haemodynamic data are presented in Table 2.

**Table 2:** Patient Basic Characteristics And Haemodynamic Data

*Patients*	*Age (months)*	*Weight (kg)*	*Height (cm)*	*Gender*	*Qp:Qs*	*Rp (WU)*	*PA systolic (mmHg)*	*Mean PA (mmHg)*
1	18	7.6	74	M	2.68	2.77	46	31
2	9	6.4	65	F	1.18	0.789	18	9
3	5	8.1	76	F	1.5	1.4	34	27
4	47	18.7	99.5	F	1.31	1.41	27	22
5	5	3.94	58	M	8.72	0.59	43	31
6	6	5.3	62	F	3.47	0.51	30	17
7	48	18.3	103	M	1.38	0.54	28	20
8	5	4.7	64	M	1.48	2.47	23	12
9	5	5.1	62	M	1.82	2.75	28	20
10	6	4.88	55.5	M	1.08	4.59	40	29
11	3	5.9	55	M	1.57	1.7	20	16
12	8	5.2	56	F	1.18	3.84	32	24
13	38	11.1	91	F	1.72	1.5	31	27
14	19	12.4	83	M	2.2	1.8	38	26
15	9	6	62	F	2.8	2.02	57	48
16	8	5.7	63	M	1.21	1.96	38	27
17	54	22.5	107.5	M	1.34	0.27	26	19
18	73	17.2	109	M	1.29	1.95	31	18
19	39	13	92.3	F	3.34	1.37	31	17
20	80	20.3	117	F	1.74	0.1	18	15
21	81	20	112	F	1.1	0.74	20	17
22	35	14.2	91.5	M	1.04	1.17	24	17
23	40	12.4	94	F	1.46	1.12	75	23
24	9	7.9	61	F	3.54	4.46	78	61
25	42	11.9	101	F	2.48	1.25	29	22
26	7	4.48	68	F	1.62	3.19	48	36
27	28	13.9	85	F	1.4	0.56	21	14
28	4	4.3	59	M	1.8	2.96	36	29
29	233	59	166	F	1.56	1.39	24	18
30	7	5.2	67	F	1.2	5.53	44	31
31	3	4.6	56	M	9.65	2.69	31	23
32	47	14.9	103	F	1.13	1.13	19	16
33	156	39.2	152	F	1.1	0.71	23	16
34	190	63	158	F	1.46	2.8	47	37
35	2	3.9	56	F	1.5	1.96	40	26
36	15	5.9	73	M	5.95	1.47	55	38
Mean					2.25	1.87	34.80	24.4

QP:QS, pulmonary blood flow:systemic blood flow ratio; RP (WU), pulmonary resistance in Wood units; PA systolic, pulmonary artery systolic pressure; Mean PA, mean pulmonary artery pressure.

The mean pulmonary artery pressure was 24.4 (SD: ± 10.4) mmHg, while the mean systolic pulmonary artery pressure was 34.8 (SD: ± 14.5) mmHg (Table 2). The Qp:Qs ratio was 2.25 (SD: ± 1.97), while the Rp mean was 1.87 (SD: ± 1.28) Wood units.

Table 3 shows angiographic data and outcomes in ductal closure using the ADO II. According to the Krichenko classification, 16 PDAs were type A (conical), four were type B (A-P window like), five were type C (tubular and more than 3 mm in length), two were type D (complex, with more than one constriction site), and nine were type E (long with sudden tapering at the pulmonary end). In terms of size, the narrowest mean ductal diameter (PDA size) was 2.74 (SD: ± 1.3) mm, with a mean PDA length of 9.5 (SD ± 4.16) and mean aortic ampulla of 9.46 (SD: ± 4.1) mm.

**Table 3:** Angiographic Data, Closure Approach And Outcomes

*Patients*	*PDA type*	*Narrowest diameter*	*PDA ampulla*	*PDA length*	*Radiation exposure*	*Mode of delivery*	*Closure device*	*Outcome*
1	A	3.6	7.4	5.6	22.4	Pulmonary	05 × 06	Immediate closure
2	A	0.6	7.8	15.3	18	Aortic	03 × 06	Immediate closure
3	A	2.2	4.78	4.08	10.21	Pulmonary	03 × 06	Closed in one month
4	A	2	10.5	8.4	17.5	Pulmonary	04 × 06	Closed on day one
5	C	2.8	4.2	6.4	21.8	Pulmonary	06 × 06	Closed on day one
6	E	1.65	6.96	7.85	21	Pulmonary	03 × 06	Lost to follow u
7	D	2	7.1	10.9	25.2	Pulmonary	06 × 06	Immediate closure
8	A	0.9	8.2	8.9	15.3	Pulmonary	03 × 06	Closed on day one
9	E	2.2	6.5	8.6	24.3	Pulmonary	04 × 06	Closed on day one
10	C	3.6	4.3	10	15.2	Pulmonary	05 × 06	Immediate closure
11	E	3.4	12.6	13.5	17.5	Pulmonary	06 × 06	Immediate closure
12	E	2.1	8.9	11.8	13.6	Pulmonary	06 × 06	Closed on day one
13	A	3	11.1	9.7	21.6	Pulmonary	06 × 06	Closed in one month
14	A	1.4	10.8	16.6	23.2	Aortic	05 × 06	Immediate closure
15	C	5.5	13.7	10.1	29.4	Pulmonary	06 × 06	Embolised, surgery
16	B	4	4.8	3.3	28.8	Pulmonary	04 × 06	Lost to follow up
17	A	2.1	12	9.2	17.5	Pulmonary	04 × 06	Closed on day one
18	A	2	12	7.4	42.1	Aortic	03 × 06	Immediate closure
19	E	4.2	15.1	14.9	14.8	Pulmonary	06 × 06	Immediate closure
20	E	3.3	17.7	17.3	7.1	Pulmonary	04 × 06	Closed on day one
21	C	1	5	8	79.21	Aortic	03 × 06	Immediate Closure
22	E	2.2	10	9.3	23.3	Pulmonary	06 × 06	Immediate Closure
23	E	1.8	5.6	11.5	88.7	Aortic	06 × 06	Immediate Closure
24	B	4	4.9	3.4	13.8	Pulmonary	04 × 04	Immediate Closure
25	D	3.5	11.8	14.5	8.5	Pulmonary	06 × 06	Immediate Closure
26	B	3	3	2.5	17	Pulmonary	04 × 04	Immediate Closure
27	A	1.86	12.56	6.07	11.1	Pulmonary	03 × 06	Closed on day one
28	A	4.5	14.6	12.7	21.2	Pulmonary	06 × 06	Immediate Closure
29	A	2.5	15.8	14.8	23.2	Pulmonary	06 × 06	Immediate Closure
30	C	3.3	7.5	15.1	14.1	Pulmonary	04 × 06	Immediate Closure
31	B	4.9	7.3	3.8	30.7	Pulmonary	06 × 04	Immediate Closure
32	A	1.8	8.6	7.6	9.7	Pulmonary	03 × 06	Immediate Closure
33	A	1.3	18.7	6	13.3	Pulmonary	04 × 06	Immediate Closure
34	A	6.2	15.4	15.6	31.5	Aortic	06 × 06	Immediate Closure
35	E	3.5	8.2	6.3	19.4	Pulmonary	05 × 06	Closed on day one
36	A	0.8	5.3	6	24.9	Pulmonary	03 × 06	Immediate Closure
Mean		2.74	9.46	9.5				

In terms of device choice, nine patients were closed using a 3 × 6-mm device, two with a 4 × 4-mm device, seven with a 4 × 6-mm device, four with a 5 × 6-mm device, one with a 6 × 4-mm device, and 13 with a 6 × 6-mm device. Regarding the delivery of the device, in 30 patients, the device was delivered through the pulmonary artery, while in six it was in a retrograde fashion through the aorta.

The exposure to radiation had a median of 20.2 minutes (range of 7.1–88.7). In terms of closure rates, 33 patients (91.67%) achieved complete closure by discharge (day one) and one additional patient by one month’s follow up. Two patients had residual PDA by three months and these patients were lost to follow up, therefore achieving a closure rate of 94.44% by three months’ follow up.

There were two patients with other congenital heart defects. One patient had a single ventricle, common atrium, pulmonary artery (PA) band, Glenn shunt and stenosis at the origin of the left and right pulmonary arteries due to the PA band. The patient had percutaneous PDA closure and right pulmonary artery-to-left pulmonary artery stenting. This patient had the longest screening time (88 minutes) as there were complications associated with the stenting of the branch pulmonary arteries. The second patient had an atrioventricular septal defect with a tiny inlet ventricular septal defect and a primum atrial septal defect, which would be attended to at a later stage.

**Fig. 3.**
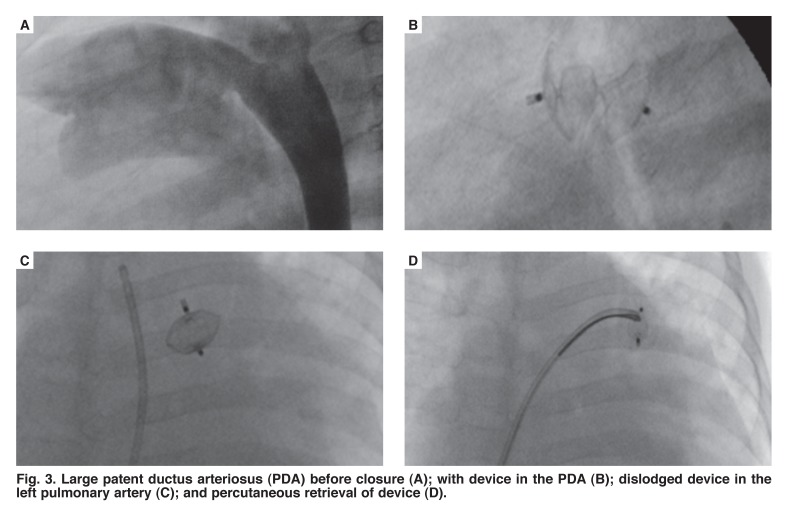
Large patent ductus arteriosus (PDA) before closure (A); with device in the PDA (B); dislodged device in the left pulmonary artery (C); and percutaneous retrieval of device (D).

When reviewing complications or outcomes; in one patient the device embolised to the left pulmonary artery following release. This device was successfully retrieved and the patient was sent for surgical closure of the PDA (Fig. 3). In another patient, there was mild left pulmonary artery (LPA) stenosis with a gradient of 15 mmHg. This gradient had not worsened on follow up. There were no other complications reported.

## Discussion

Percutaneous closure of symptomatic PDA has become the preferred method over surgical closure. For moderate to large (> 3 mm) PDAs, the Amplatzer duct occludder type I has been the device of choice.^15^ However, there are limitations in using the ADO I device. The device is made of polyester material, which makes it cumbersome and it requires a large delivery system (5–7 F) (St Jude Medical, Cardiovascular Division, St Paul, MN). This large delivery system makes it difficult and rather challenging to close a moderate to large PDA in small infants (< 6 kg). The device may also cause coarctation of the aorta due to its large retention skirt, which is on the aortic side.^19^

While the ADO II has a low profile and low delivery system, it can also cause aortic and left pulmonary artery (LPA) obstruction like the ADO I.^20^-^22^ In this series, there was one patient who had mild LPA obstruction. Both devices have the potential to embolise.^21^

We had one device that embolised. The patient (patient 15) had a large PDA with its narrowest diameter being 5.5 mm, which is the upper limit for percutaneous closure using ADO II, according to the manufacture’s guidelines (see Table 1). This patient had a large left-to-right shunt with a Qp:Qs ratio of 2.8:1. The duct morphology itself was more tubular than conical. The ductal size and the shape of the PDA were high risk factors for embolisation in this patient. The device was successfully retrieved and the duct was deemed unsuitable for percutaneous closure and as a result was closed surgically.

Care should be taken when choosing a device for closure of large PDAs with less suitable anatomy, as in such patients, the device might embolise. There were no major catheterisation-related complications in this study, such as bleeding, requiring blood transfusion, loss of femoral arterial pulse or arterial avulsion, as reported elsewhere.^21^,^22^

The low-profile TorqVue delivery system of the ADO II (4–5 F) allows this device to be used to close PDAs in smaller infants (< 6 kg) with a limited risk of causing either aortic or pulmonary obstruction in carefully selected patients. In this series, there were 10 patients weighing less than 6 kg who underwent ductal closure using the ADO II, contrary to the manufacturer’s recommendations. This device was also able to close ducts in patients less than six months of age. Eight patients in this study were younger than six months of age (range 2–5 months).

Another advantage of this device over the ADO I is that it may be introduced both in anterograde fashion through the pulmonary side and in retrograde approach through the arterial side to close the PDA. In this report, there were six patients who had their ducts closed through the arterial side. Except for one patient whose duct was 6.2 mm, PDAs less than 3 mm (range 0.6–2 mm) were closed through the aortic route.

Historically, smaller PDAs (≤ 3 mm) would be closed with Cook’s or Gianturco coils or the Nit Occlud device.^21^ The ADO II has offered an alternative to this mode of closure. It should be mentioned though that the ADO II remains more expensive than the Gianturco coils. It has also been shown that the coils have less screening time and have shown less use of contrast than the Amplatzer device.^21^

**Fig. 4.**
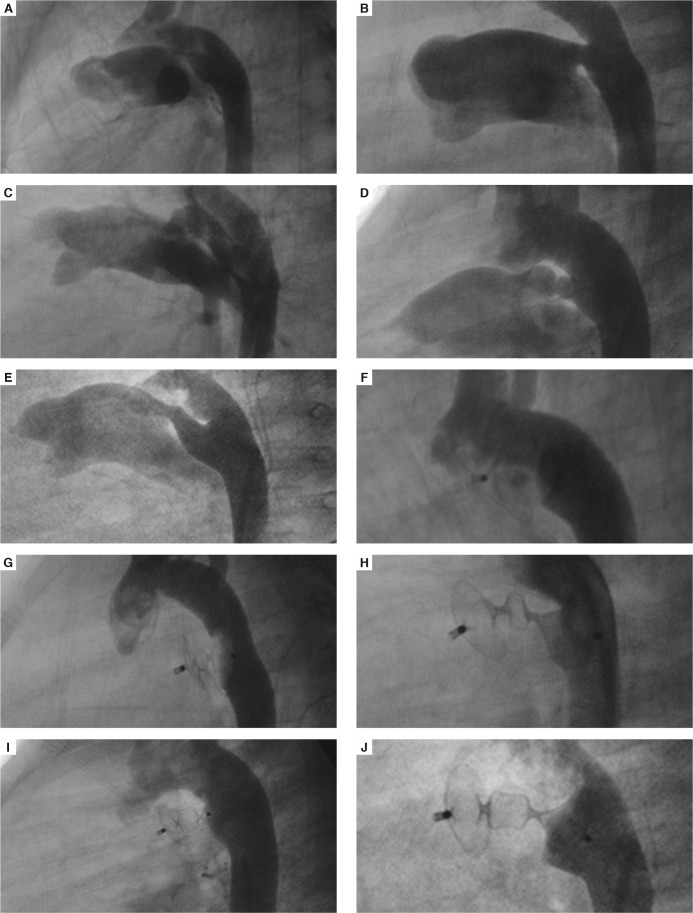
Descending aortograms in straight lateral view, showing Krichenko type A, B, C, D, and E PDAs before closure (A-E) and after closure (F-J) with the Amplatzer duct occluder type II. (Figures A and F, patient 13; B and G, patient 24; C and H, patient 10, D and I, patient 7; E and J patient 12).

When it comes to ductal shape, other devices such as the ADO I, coils and Nit Occlud device would close Krichenko type A (conical) PDAs. The ADO II has been shown in this report (Fig. 4) and others to be able to close all anatomical types of PDAs, including tubular and long (type C), and tubular, AP-window like ducts with a shallow aortic ampulla (type B).^17^,^20^,^21^ Two patients had residual ducts at three months of follow up. Residual ducts have been reported in other studies.^23^,^24^ These were closed using coils or the Nit Occlud device.

In terms of the narrowest diameter of the PDA, the manufacturer recommends closure of the PDA using the ADO II up to 5.5 mm. In this series, there was one patient who had a PDA that measured 6.2 mm, which was 0.7 mm more than the recommended upper limit. In favour of percutaneous closure in this patient was the shape of the PDA, which was more conical with a larger ampulla of 15.4 mm. This patient also had a small left-to-right shunt of 1.46:1. This may have been due to the fact that the patient had significant pulmonary hypertension with a pulmonary artery mean of 37 mmHg, thus limiting left-to-right shunting across the PDA. The duct was amenable to percutaneous closure though, as the Rp was high-normal at 2.8 Wood units.

The mean screening time of 23.4 (± 16.66) min was longer than in other studies.^17^,^22^ Limitations to the use of this device would include inability to close a very large PDA, as the largest size is 6 × 6 mm, with a retention disk of 12 mm; and inability to occlude a duct with a shallow ampula and a small aorta or pulmonary artery, as closure in such patients might cause significant aortic coarctation or left pulmonary artery stenosis. The introduction of ADO II additional sizes and the use of an Amplatzer vascular plug II for ductal closure, which has a much smaller profile with smaller retention disks, has offered hope for closure of PDAs in much smaller infants, including newborns.^21^,^25^,^26^

The major limitation of this study was that this was a retrospective analysis of records. There was no direct comparison between this device and other devices used for percutaneous ductal occlusion, including the ADO I.

## Conclusion

The Amplatzer duct occluder II is able to close all types of PDAs in very small infants (< 6 kg). The device may be utilised to close PDAs historically closed using coils. Its ability to be delivered via both pulmonary and aortic approaches expands its use, including patients whose anatomy is difficult to approach either through the pulmonary side or the aorta.
